# COVID-19 symptom duration: associations with age, severity and vaccination status in Brunei Darussalam, 2021

**DOI:** 10.5365/wpsar.2022.13.4.941

**Published:** 2022-11-07

**Authors:** Shi Ying Tan, Shyh Poh Teo, Muhd Syafiq Abdullah, Pui Lin Chong, Rosmonaliza Asli, Babu Ivan Mani, Natalie Riamiza Momin, Adrian Chin Ann Lim, Noor Affizan Rahman, Chee Fui Chong, Vui Heng Chong

**Affiliations:** a National Isolation Centre, Ministry of Health, Tutong, Brunei Darussalam.; b Department of Medicine, Raja Isteri Pengiran Anak Saleha Hospital, Bandar Seri Begawan, Brunei Darussalam.; c Pengiran Anak Puteri Rashidah Sa’adatul Bolkiah Institute of Health Sciences, Universiti Brunei Darussalam, Bandar Seri Begawan, Brunei Darussalam.

## Abstract

**Objective:**

This retrospective, cross-sectional, observational study assessed the duration of coronavirus disease 2019 (COVID-19) symptoms during the second wave in Brunei Darussalam.

**Methods:**

Data from COVID-19 cases admitted to the National Isolation Centre during 7–30 August 2021 were included in the study. Symptom onset and daily symptom assessments were entered into a database during hospitalization and disease was categorized by severity. The time between symptom onset and hospital admission, the duration of symptoms and length of hospitalization were assessed separately by age group, disease severity and vaccination status using one-way analysis of variance with Bonferroni post hoc corrections.

**Results:**

Data from 548 cases were included in the study: 55.7% (305) of cases were male, and cases had a mean age of 33.7 years. Overall, 81.3% (446) reported symptoms at admission (mean number of symptoms and standard deviation: 2.8 ± 1.6), with cough (59.1%; 324), fever (38.9%; 213) and sore throat (18.4%; 101) being the most common. Being older, having more severe disease and being unvaccinated were significantly associated with the time between symptom onset and hospital admission, symptom duration and length of hospitalization.

**Discussion:**

Knowing which factors predict the duration of COVID-19 symptoms can help in planning management strategies, such as the duration of isolation, predict the length of hospitalization and treatment, and provide more accurate counselling to patients regarding their illness.

The coronavirus disease 2019 (COVID-19) pandemic, caused by infection with severe acute respiratory syndrome coronavirus 2 (SARS-CoV-2), continues, bringing significant morbidity and mortality. As of 15 September 2022, there were more than 607 million confirmed cases and nearly 6.5 million deaths worldwide. (*1*) Since the pandemic began, there has been a rapid increase in understanding of the disease and its management, with swift development and approval of vaccines and therapeutics. In Brunei Darussalam, the first wave of the COVID-19 pandemic started on 9 March 2020 with the detection of the first case, a patient who had attended a religious gathering in a neighbouring country. The last community case was detected on 6 May 2020, with a total of 141 cases reported at that point. Only imported cases were detected until the second wave started on 7 August 2021, during which the predominant SARS-CoV-2 strain was confirmed to be Delta (B.1.617.2). (*2*) The third wave, caused by the more infectious Omicron strain, started on 5 February 2022. (*3*)

COVID-19 is predominantly a respiratory illness. Common symptoms include fever, cough, fatigue, body aches, sore throat, anosmia and ageusia/dysgeusia. (*4*) Severe symptoms include shortness of breath and chest pain. Symptoms may appear 2–14 days after exposure and usually last 5–7 days, but they can be prolonged, resulting in more severe illness. Clinically, COVID-19 ranges from an asymptomatic presentation to severe pneumonia requiring ventilatory support and causing death. Nonrespiratory symptoms may also occur, including cardiovascular, gastrointestinal, neurological and cutaneous symptoms. (*5*) In earlier reports from China, anosmia and ageusia were not recognized as typical COVID-19 symptoms, but they are currently acknowledged as distinctive symptoms. (*6*-*8*) Differences in symptoms and their duration may be due to viral variants, underlying comorbidities or race, or a combination of these.

While the symptoms of COVID-19 are well known, the duration of symptoms is less well studied. This study aimed to assess the duration of symptoms of COVID-19 by age, disease severity and vaccination status in Brunei Darussalam during the start of the second wave of the pandemic.

## Methods

### Study design and population

This was a retrospective, cross-sectional, observational study conducted using data from cases diagnosed with SARS-CoV-2 infection by reverse transcription–polymerase chain reaction (RT–PCR) who were admitted to the National Isolation Centre (NIC) in Brunei Darussalam, between 7 August 2021 (the start of the second wave) and 30 August 2021. Individuals with incomplete records were excluded from the study.

### Setting and management

In Brunei Darussalam, all cases diagnosed with COVID-19 were admitted to the NIC for isolation and treatment. This arrangement continued until the second week of the second wave (18 August 2021), when community isolation centres were opened to cope with the increasing number of cases. During admission to the NIC, patients provided a detailed history of symptoms and underwent clinical examination and relevant investigations, such as laboratory testing and chest imaging. During hospitalization, cases had their symptoms assessed and documented daily. Cases underwent an RT–PCR test on day 8 to determine if they could be discharged, and they were considered recovered if this test was negative. If the day 8 test was positive, RT–PCR was repeated at 48-hour intervals. A case was considered recovered if the day 10 or subsequent test was negative or a cycle threshold value > 30.0 was obtained.

### Disease severity categories

Cases were categorized based on disease severity as follows: category 1 (C1) – asymptomatic; C2 – symptomatic but without pneumonia (clinical or radiological); C3 – pneumonia; C4 – needing oxygen therapy; and C5 – needing intubation and ventilatory support, with or without other organ failure. Category 2 is divided into two subcategories: C2a – milder symptoms (i.e. cough, nausea, vomiting, rhinorrhoea, anosmia or dysosmia, ageusia or dysgeusia, diarrhoea < 2 times in 24 hours, myalgia and lethargy); and C2b – worsening C2a symptoms (i.e. new onset fever, fever persistence > 2 days, chest pain, dyspnoea, unable to ambulate independently, reduced oral intake and reduced urine output). These categories were introduced and implemented on 13 August 2021. (*9*)

### Symptom categories

Cases were categorized based on their reported symptoms at admission: asymptomatic (no symptoms), presymptomatic (asymptomatic at admission but subsequently developed symptoms during hospitalization; these cases were reassigned at hospital discharge), recovered (symptoms had resolved by admission) and symptomatic (symptomatic at admission).

### Data collection

Details of cases and the relevant investigations were collected and prospectively entered into a database. This database was used to track patients’ movements between the NIC, community isolation centres and home; track clinical progress; assist in the overall management of COVID-19 cases; and report daily to the Ministry of Health. Data collected included age, sex, vaccination status, symptoms reported, disease severity, and prevalence and duration of oxygen use.

Symptom duration was calculated based on the reported date of symptom onset, with symptom resolution considered to be the first day the case achieved disease category C1.

### Vaccination status

The Brunei Darussalam national vaccination programme for COVID-19 was implemented in phases beginning 3 April 2021; the first to be vaccinated were frontline staff and older people, followed by people with comorbidities. (*10*) The COVID-19 vaccines used were BBIBP-CorV (Sinopharm), Comirnaty (Pfizer–BioNTech), Spikevax (Moderna) and Vaxzevria (Oxford–AstraZeneca). Vaccination status was categorized as complete (received two doses), partial (received one dose), unvaccinated and ineligible (age < 18 years or had any contraindication based on recommendations at the time, such as receiving chemotherapy or on immunosuppressive medications). A vaccine dose was considered complete if the case had received it at least 14 days before COVID-19 infection. At the time of the study, COVID-19 vaccine booster doses (third doses) had not yet been introduced.

### Statistical analysis

Anonymized data were entered into the database and analysed using IBM SPSS version 26.0 (IBM, New York, United States of America). Descriptive statistics were used to describe case characteristics. Age was divided into several categories: £12 years (children), 13–19 years, 20–29 years, 30–39 years, 40–49 years, 50–59 years and (*3*)60 years. One-way analysis of variance (ANOVA) testing was used to assess the time between symptom onset and hospital admission, symptom duration and length of hospitalization separately by age group, disease severity and vaccination status. This provided *P*-values for trends. Post hoc Bonferroni analysis was used to assess the differences between the age groups, again providing *P*-values for each comparison. A scatterplot was used to assess correlations between age and symptom duration. Two-sided tests were used and *P* < 0.05 was considered statistically significant.

## Results

Of the 751 COVID-19 cases admitted to the NIC during the study period, 548 (73%) were included in the analysis and 203 cases were excluded. Reasons for exclusion were being admitted before use of the disease severity score (163 cases), death (20) and incomplete data (20).

The mean age of COVID-19 cases included in the analysis was 33.7 years; there was a higher proportion of males (55.7%; 305). The most common comorbidities were hypertension (18.1%; 99), dyslipidaemia (10.4%; 57) and diabetes mellitus (9.5%; 52). Most cases were either unvaccinated (59.1%; 324) or ineligible for vaccination because of age (20.8%; 114), with only 13.0% (71) having had at least one dose of vaccine (**Table 1**).

**Table 1:** Characteristics of 548 COVID-19 cases admitted to the National Isolation Centre, 7–30 August 2021, Brunei Darussalam

Characteristic	No. (%)
Age group (years)	
£12	64 (11.7)
13–19	55 (10.0)
20–29	118 (21.5)
30–39	101 (18.4)
40–49	111 (20.3)
50–59	59 (10.8)
^3^60	40 (7.3)
Sex	
Male	305 (55.7)
Female	243 (44.3)
Comorbidities	
Diabetes mellitus	52 (9.5)
Dyslipidaemia	57 (10.4)
Hypertension	99 (18.1)
Vaccination status	
Ineligible	114 (20.8)
Unvaccinated	324 (59.1)
Partial (1 dose)	71 (13.0)
Complete (2 doses)	39 (7.1)

Symptoms were reported at admission by 81.3% (446) of cases, with the most common being cough (59.1%; 324), fever (38.9%; 213) and sore throat (18.4%; 101) (**Table 2**). Among these symptomatic cases, the mean number of symptoms reported on admission was 2.8. There was no significant difference in the number of symptoms reported by disease category (given with the standard deviation [SD]) as classified during hospitalization: C2 = 2.8 ± 1.6; C3 = 2.8 ± 1.6; C4 = 3.1 ± 1.6; and C5 = 3.0 ± 0.9 (*P* = 0.065 by ANOVA).

**Table 2:** Reported symptoms in 548 COVID-19 cases admitted to the National Isolation Centre, 7–30 August 2021, Brunei Darussalam

Symptom	No. (%)
Symptoms reported at admission	446 (81.3)
Cough	324 (59.1)
Fever	213 (38.9)
Sore throat	101 (18.4)
Rhinorrhoea	97 (17.7)
Anosmia	86 (15.7)
Dyspnoea	74 (13.5)
Ageusia or dysgeusia	70 (12.8)
Loose stool or diarrhoea	54 (9.9)
Myalgia	48 (8.8)
Headache	41 (7.5)
Nausea or vomiting	19 (3.5)
Symptom category	
Asymptomatic	73 (13.3)
Recovered	40 (7.3)
Presymptomatic	29 (5.3)
Symptomatic	406 (74.1)
Disease category at admission	
C1	187 (34.1)
C2	315 (57.5)
C3	13 (2.4)
C4	33 (6.0)
C5	0
Most severe disease category during hospitalization	
C1	113 (20.6)
C2	267 (48.7)
C3	95 (17.3)
C4	63 (11.5)
C5	10 (1.8)

C1: asymptomatic; C2: symptomatic but without pneumonia (clinical or radiological); C3: pneumonia; C4: needing oxygen therapy; C5: needing intubation and ventilatory support with or without other organ failure.

**Fig. 1:**
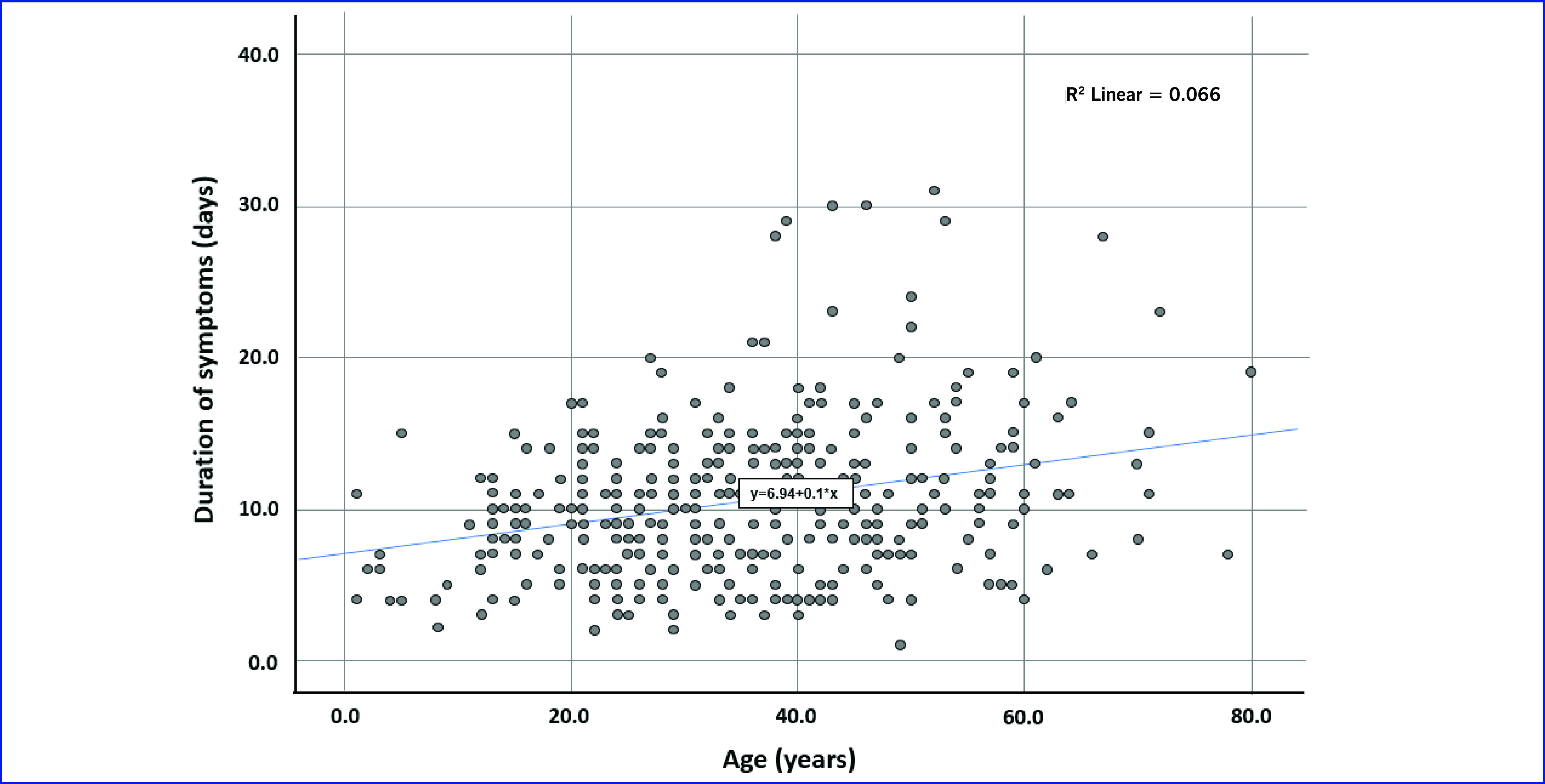
Scatterplot of age and duration of COVID-19 symptoms for 548 cases admitted to the National Isolation Centre, 7–30 August 2021, Brunei Darussalam

The mean (SD) number of days between symptom onset and admission was 4.9 (± 3.4). The mean (SD) symptom duration was 10.4 (± 5.1) days. The mean (SD) length of hospitalization was 10.8 (± 4.3) days. There was a positive correlation between age and symptom duration, with a predicted increase in symptom duration of 0.1 day for each additional year increase in age  (y = 6.94 + 0.1x) (**Fig. 1**). Each of these categories was also significantly different by age group (*P* = 0.034 for symptom onset to admission, *P* < 0.001 for symptom duration, *P* = 0.004 for length of hospitalization; **Table 3**).

[**insert** Figure 1]

**Table 3:** Time from symptom onset to admission, and duration of symptoms and hospitalization, by age group and disease category for 548 COVID-19 cases admitted to the National Isolation Centre, 7–30 August 2021, Brunei Darussalam

Characteristic	Time from symptom onset to admission (days)	*P*	Duration of symptoms (days)	*P*	Duration of hospitalization (days)	*P*
Age group (years)						
£12	3.5 ± 2.5^a^	0.034	6.9 ± 3.3^b^	< 0.001	10.9 ± 3.3	0.004
13–19	4.1 ± 2.3	-	8.8 ± 2.8^c^	-	9.1 ± 3.3^e^	-
20–29	4.8 ± 3.3	-	9.2 ± 4.2^d^	-	10.4 ± 3.6	-
30–39	5.0 ± 3.3	-	10.7 ± 4.9	-	10.5 ± 4.0	-
40–49	4.9 ± 3.1	-	10.9 ± 5.6	-	11.5 ± 5.3	-
50–59	6.2 ± 4.7	-	12.6 ± 6.3	-	11.5 ± 4.4	-
^3^60	4.4 ± 2.9	-	13.3 ± 5.9	-	12.5 ± 5.6	-
Disease category						
C1	NA	0.018	NA	< 0.001	9.2 ± 4.2^j^	< 0.001
C2	4.4 ± 3.0^f^	-	8.7 ± 3.9^g^	-	10.1 ± 3.5^k^	-
C3	5.7 ± 3.5	-	11.4 ± 4.8^h^	-	11.7 ± 4.1^l^	-
C4	4.6 ± 3.9	-	16.1 ± 6.0^i^	-	14.9 ± 4.9^m^	-
C5	5.8 ± 3.3	-	NA	-	NA	-
Overall	4.8 ± 3.3	-	10.4 ± 5.1	-	10.8 ± 4.3	-

Values are mean ± SD

C1: asymptomatic; C2: symptomatic but without pneumonia (clinical or radiological); C3: pneumonia; C4: needing oxygen therapy; C5: needing intubation and ventilatory support with or without other organ failure; NA: not applicable; SD: standard deviation.

^a^ Significant compared with 50–59 year age group (*P* = 0.045).

^b^ Significant compared with 50–59 year age group (*P* = 0.002) and (*3*)60 year age group (*P* = 0.002).

^c^ Significant compared with 50–59 year age group (*P* = 0.019) and (*3*)60 year age group (*P* = 0.022).

^d^ Significant compared with 50–59 year age group (*P* = 0.004) and (*3*)60 year age group (*P* = 0.012).

^e^ Significant compared with 40–49 year age group (*P* = 0.019) and (*3*)60 year age group (*P* = 0.006).

^f^ Comparison between C2 and C3 categories (*P* = 0.017).

^g^ Significant compared with C3 and C4 categories (both *P* < 0.001).

^h^ Significant compared with C2 and C4 categories (both *P* < 0.001).

^i^ Significant compared with C2 and C3 categories (both *P* < 0.001).

^j^ Significant compared with C3 and C4 categories (both *P* < 0.001).

^k^ Significant compared with C3 category (*P* = 0.005) and C4 (*P* < 0.001).

^l^ Significant compared with C1 category (*P* < 0.001), C2 (*P* = 0.005) and C4 (*P* < 0.001).

^m^ Significant compared with C1, C2 and C3 categories (all *P* < 0.001).

When comparing each of these by age group, for time of symptom onset to admission, the youngest age group (£12 years) had a significantly shorter interval compared with those in the 50–59 year group. There were no differences in this category between any of the other age groups. Symptom duration was significantly shorter for each of the three youngest age groups (£12, 13–19 and 20–29 years) when compared with each of the two oldest age groups (50–59 and (*3*)60 years). Length of hospitalization was significantly shorter in adolescents (13–19 years) compared with those in the 40–49 year group, with all other comparisons being non-significant (**Table 3**).

There was a statistically significant difference between each of these categories and the disease category (*P* < 0.001 each for time from symptom onset to admission, symptom duration and length of hospitalization). When comparing each of these categories with the disease category groups, C2 cases had a significantly shorter interval from symptom onset to admission when compared with C3 cases; symptom duration was significantly shorter in C2 cases when compared with C3 and C4 cases; and length of hospitalization was significantly shorter for the less severe category for most comparisons between C1, C2, C3 and C4 cases (**Table 3**).

Altogether, 6.0% (33) of cases were categorized as C4 at admission and 11.5% (63) were assessed as C4 at any time during their hospitalization. For the 63 cases assessed as C4 at any time during their illness, the mean (SD) interval from symptom onset to admission was 4.6 (± 3.9) days, and the mean (SD) interval from admission to needing oxygen therapy was 2.1 (± 2.5) days. For all cases, the mean (SD) interval from symptom onset to needing oxygen was 6.8 (± 3.9) days. For all cases, the mean (SD) duration of oxygen therapy was 6.4 (± 5.4) days.

Cases who had received two doses of COVID-19 vaccine had a significantly shorter symptom duration than cases who had received one dose of vaccine or were unvaccinated. Those ineligible for vaccination had a significantly shorter symptom duration compared with unvaccinated cases (**Table 4**).

**Table 4:** Duration of COVID-19 symptoms by vaccination status for 548 cases admitted to the National Isolation Centre, 7–30 August 2021, Brunei Darussalam

Vaccination status	Duration of symptoms (days, mean ± SD)	*P*
Overall	10.4 ± 5.1	< 0.001
Complete	6.8 ± 3.5^a^	
Partial	10.1 ± 5.2	
Unvaccinated	11.3 ± 5.3	
Ineligible	8.2 ± 3.1^b^	

SD: standard deviation.

^a^ Significant compared with partial (*P* = 0.024) and unvaccinated (*P* < 0.001).

^b^ Significant compared with unvaccinated (*P* < 0.001).

## Discussion

This study showed that the duration of COVID-19 symptoms was associated in separate analyses with being older, having more severe disease and being unvaccinated. Younger cases had a shorter duration of symptoms compared with older cases; cases with less severe disease (C1 and C2) had a shorter duration of symptoms than those with more severe disease (C3 and C4); and those who were fully vaccinated had a shorter duration of symptoms than those who were unvaccinated. Knowing about symptoms, including their likely duration, can help in planning management strategies, such as the duration of isolation or quarantine, predicting the length of hospitalization and treatment, as well as providing better and more accurate counselling to patients regarding COVID-19, depending on the severity of their disease at the time of presentation. This information may also encourage cases to present in a timely manner if their illness does not improve as expected, particularly those who have not been admitted or are isolating at home.

The mean (SD) duration of symptoms of COVID-19 in this study was 10.4 (± 5.1) days, with the duration of symptoms increasing approximately linearly with age. Symptom duration was associated with age group, with younger cases having shorter duration. Generally, symptomatic children have mild disease and a short duration of illness, (*11*, *12*) with one study from the United Kingdom of Great Britain and Northern Ireland reporting that the median duration of illness was shorter for younger children (duration: 5 days, interquartile range [IQR] = 2–9) compared with older children (duration: 7 days, IQR = 3–12). (*12*) A study from Italy reported that 4.4% of children had prolonged illness lasting up to 28 days, and this was more common in older children (5.1%) than in younger children (3.1%) (*P* = 0.046). (*13*) A study from the United States of America reported that at least one in five young, healthy adults aged 18–34 years had unresolved symptoms up to 3 weeks after diagnosis. (*14*)

Symptom duration has also been correlated with the duration of viral shedding and infectivity, especially during the first 2 weeks of symptoms. (*15*, *16*) Knowledge of symptom duration may be useful as a proxy measure for infectivity in patients, removing the need for laboratory testing. This is important for advising patients about the required period of isolation or quarantine, and it also applies to asymptomatic cases, as studies have shown no differences in clinical features and virological course in cases with asymptomatic or symptomatic non-severe COVID-19. (*17*)

Apart from the correlation with age, this study also showed that symptom duration was associated with disease severity. Cases in the C2 symptomatic category had a mean (SD) duration of symptoms of 8.7 (± 3.9) days. This was significantly shorter than in those who had pneumonia on imaging (C3: 11.4 ± 4.8 days) and those needing oxygen therapy (C4: 16.1 ± 5.1 days). This is expected: the more severe the illness, the longer it would take to recover. To date, no studies have assessed symptom duration based on the severity of disease. Viral shedding has been shown to correlate with symptom duration and severity of illness. (*18*)

This study also showed that vaccination status was associated with symptom duration: fully vaccinated cases had a significantly shorter duration of symptoms than unvaccinated and partially vaccinated cases. Vaccination reduces the risk of COVID-19, (*19*-*22*) as well as the duration and severity of illness. (*23*) In the United Kingdom, vaccinated cases were more likely to be asymptomatic, had fewer symptomatic days and less severe illness, and had lower hospitalization rates, with the analysis including patients aged (*3*)60 years. (*24*) The impact of vaccination on symptom duration should reinforce the drive to vaccinate as many people as possible during the current pandemic. While the first vaccine dose provides some protection, completing the two-dose primary vaccination series provides better protection against infection. (*25*) This study showed that cases who had received a two-dose vaccination regimen had shorter duration of symptoms compared with unvaccinated and partially vaccinated cases.

Among cases who required oxygen therapy (C4), the requirement for oxygen occurred a mean (SD) of 6.8 (± 3.9) days after symptom onset. The mean (SD) duration of oxygen therapy was 6.4 (± 5.4) days, similar to cases in Ethiopia (6.0 days), (*26*) but shorter than patients in Germany (8.0 days). (*27*) This may be due to the relatively high proportion of young cases in this study. Older cases required a longer time to be weaned off oxygen therapy due to their comorbidities and reduced immunity. (*26*) With the Delta variant of COVID-19, there is a rapid transition from becoming symptomatic to having dyspnoea and needing oxygen therapy, perhaps exacerbated by the phenomenon of happy (or silent) hypoxia. (*28*, *29*) For cases admitted to hospital, pneumonia can be identified early by the deterioration in their condition and with chest imaging so that the need for oxygen therapy can be anticipated. However, knowing the average time from symptom onset to oxygen requirement can be useful to gauge when to closely monitor cases at risk of further deterioration.

There are several limitations to this study. First, while it was a retrospective study, it used prospectively collected data from a real-time database used for patient management. Retrospective studies are associated with missing or incomplete data. Even though the data used in this study were captured prospectively, some data were missing due to the number of cases; thus, a small number had to be excluded. Second, this study included cases from the first few weeks of the second wave of the pandemic, when all cases were admitted to the NIC. This enabled the whole spectrum of COVID-19 disease severity to be studied, but it meant that cases occurring after this time were excluded. In addition, cases admitted during the first 6 days of the second wave were excluded from the study, as disease was categorized differently. (*9*) Third, symptoms were closely followed only during hospitalization; thus, cases with mild to moderate disease (C1 or C2) or symptoms persisting after discharge were not evaluated. However, using the length of hospitalization was deemed to be adequate to cover the duration of illness, taking into account the interval from symptom onset to admission. Additionally, management protocols permit patients to be discharged only after clinical improvement, with most being fully recovered on discharge.

The main strength of this study is its link to the patient management system that required all cases to be assessed daily and included information about their symptoms, and this daily assessment was continued for the duration of hospitalization. This allowed accurate data to be collected systematically, which was possible due to the local management protocol requiring cases to be asymptomatic or minimally symptomatic before repeating SARS-CoV-2 testing to document recovery. Furthermore, this study also assessed the association between symptom duration and vaccination status, information that has not been published previously.

In conclusion, this study showed that symptom duration was associated separately with age, disease severity and vaccination status, with longer duration of symptoms associated with being older and having more severe disease. Receiving two doses of COVID-19 vaccine was significantly associated with a shorter duration of symptoms, highlighting the importance of vaccination. These findings are relevant as they illustrate that the duration of symptoms varied and was affected by several factors. Recommendations about the duration of isolation for patients who do not require hospitalization, discharge planning and counselling of patients diagnosed with COVID-19 can be guided by this information. This is mostly relevant for cases infected with the Delta strain of SARS-CoV-2, but it may also provide a reference for other variants that may emerge during the pandemic.

## References

[1] WHO coronavirus (COVID-19) dashboard. Geneva: World Health Organization; 2020. Available from: https://covid19.who.int/, accessed 16 September 2022.

[2] 106 new cases COVID-19 reported today, 26 August 2021: media statement on the current situation of COVID-19 in Brunei Darussalam. Bandar Seri Begawan: Ministry of Health; 2021. Available from: https://www.moh.gov.bn/Lists/Latest%20news/NewDispForm.aspx?ID=1007, accessed 14 April 2022.

[3] 368 new cases COVID-19 reported today, 07 February 2022: media statement on the current situation of COVID-19 in Brunei Darussalam. Bandar Seri Begawan: Ministry of Health; 2022. Available from: https://www.moh.gov.bn/Lists/Latest%20news/NewDispForm.aspx?ID=1159, accessed 14 April 2022.

[4] Symptoms of COVID-19. Atlanta (GA): Centers for Disease Control and Prevention; 2022. Available from: https://www.cdc.gov/coronavirus/2019-ncov/symptoms-testing/symptoms.html, accessed 14 April 2022.

[5] Mizrahi B, Shilo S, Rossman H, Kalkstein N, Marcus K, Barer Y, et al. Longitudinal symptom dynamics of COVID-19 infection. Nat Commun. 2020 12 4;11(1):6208. 10.1038/s41467-020-20053-y 33277494 PMC7718370

[6] Santos REA, da Silva MG, do Monte Silva MCB, Barbosa DAM, Gomes ALDV, Galindo LCM, et al. Onset and duration of symptoms of loss of smell/taste in patients with COVID-19: A systematic review. Am J Otolaryngol. 2021 Mar-Apr;42(2):102889. 10.1016/j.amjoto.2020.102889 33445036 PMC7833280

[7] Lechien JR, Chiesa-Estomba CM, De Siati DR, Horoi M, Le Bon SD, Rodriguez A, et al. Olfactory and gustatory dysfunctions as a clinical presentation of mild-to-moderate forms of the coronavirus disease (COVID-19): a multicenter European study. Eur Arch Otorhinolaryngol. 2020 Aug;277(8):2251–61. 10.1007/s00405-020-05965-1 32253535 PMC7134551

[8] Sheng WH, Liu WD, Wang JT, Chang SY, Chang SC. Dysosmia and dysgeusia in patients with COVID-19 in northern Taiwan. J Formos Med Assoc. 2021 Jan;120(1 Pt 2):311–7. 10.1016/j.jfma.2020.10.003 33139151 PMC7574720

[9] Rahman AR, Abdullah MA, Asli R, Chong PL, Mani BI, Chong VH. Challenges during the second wave of COVID-19 in Brunei Darussalam: National Isolation Centre to national COVID-19 hospital. West Pac Surveill Response. 2022;13(3):1–7. 10.5365/wpsar.2022.13.3.913 PMC9831601 36688181

[10] The National Vaccination Program for COVID-19 commenced on Saturday, 20 Syaaban 1442/3 April 2021 and will be implemented in phases outlined in the National Vaccination Program for Brunei Darussalam. Bandar Seri Begawan: Ministry of Health; 2021. Available from: https://www.moh.gov.bn/SitePages/COVID-19%20Vaccine.aspx, accessed 15 April 2022.

[11] Viner RM, Ward JL, Hudson LD, Ashe M, Patel SV, Hargreaves D, et al. Systematic review of reviews of symptoms and signs of COVID-19 in children and adolescents. Arch Dis Child. 2020:archdischild-2020–320972. doi:10.1136/archdischild-2020-320972 pmid:33334728. 10.1136/archdischild-2020-320972 33334728

[12] Molteni E, Sudre CH, Canas LS, Bhopal SS, Hughes RC, Antonelli M, et al. Illness duration and symptom profile in symptomatic UK school-aged children tested for SARS-CoV-2. Lancet Child Adolesc Health. 2021 Oct;5(10):708–18. 10.1016/S2352-4642(21)00198-X 34358472 PMC8443448

[13] Vassallo M, Manni S, Klotz C, Fabre R, Pini P, Blanchouin E, et al. Patients admitted for variant alpha COVID-19 have poorer outcomes than those infected with the old strain. J Clin Med. 2021 08 12;10(16):3550. 10.3390/jcm10163550 34441844 PMC8396910

[14] Tenforde MW, Kim SS, Lindsell CJ, Billig Rose E, Shapiro NI, Files DC, et al.; IVY Network Investigators; CDC COVID-19 Response Team; IVY Network Investigators. Symptom duration and risk factors for delayed return to usual health among outpatients with COVID-19 in a multistate health care systems network – United States, March–June 2020. MMWR Morb Mortal Wkly Rep. 2020 07 31;69(30):993–8. 10.15585/mmwr.mm6930e1 32730238 PMC7392393

[15] Kim DY, Bae EK, Seo JW, Yun NR, Kim CM, Kim DM. Viral kinetics of severe acute respiratory syndrome coronavirus 2 in patients with coronavirus disease 2019. Microbiol Spectr. 2021 Oct 31;9(2):e0079321. 10.1128/Spectrum.00793-21 34704783 PMC8549742

[16] van Kampen JJA, van de Vijver DAMC, Fraaij PLA, Haagmans BL, Lamers MM, Okba N, et al. Duration and key determinants of infectious virus shedding in hospitalized patients with coronavirus disease-2019 (COVID-19). Nat Commun. 2021 01 11;12(1):267. 10.1038/s41467-020-20568-4 33431879 PMC7801729

[17] Li Y, Shi J, Xia J, Duan J, Chen L, Yu X, et al. Asymptomatic and symptomatic patients with non-severe coronavirus disease (COVID-19) have similar clinical features and virological courses: a retrospective single center study. Front Microbiol. 2020 06 26;11:1570. 10.3389/fmicb.2020.01570 32754137 PMC7344298

[18] Munker D, Osterman A, Stubbe H, Muenchhoff M, Veit T, Weinberger T, et al. Dynamics of SARS-CoV-2 shedding in the respiratory tract depends on the severity of disease in COVID-19 patients. Eur Respir J. 2021 07 20;58(1):2002724. 10.1183/13993003.02724-2020 33602859 PMC7898160

[19] Voysey M, Clemens SAC, Madhi SA, Weckx LY, Folegatti PM, Aley PK, et al.; Oxford COVID Vaccine Trial Group. Safety and efficacy of the ChAdOx1 nCoV-19 vaccine (AZD1222) against SARS-CoV-2: an interim analysis of four randomised controlled trials in Brazil, South Africa, and the UK. Lancet. 2021 Jan 9;397(10269):99–111. 10.1016/S0140-6736(20)32661-1 33306989 PMC7723445

[20] Baden LR, El Sahly HM, Essink B, Kotloff K, Frey S, Novak R, et al.; COVE Study Group. Efficacy and safety of the mRNA-1273 SARS-CoV-2 vaccine. N Engl J Med. 2021 Feb 4;384(5):403–16. 10.1056/NEJMoa2035389 33378609 PMC7787219

[21] Thomas SJ, Moreira ED Jr, Kitchin N, Absalon J, Gurtman A, Lockhart S, et al.; C4591001 Clinical Trial Group. C4591001 Clinical Trial Group. Safety and efficacy of the BNT162b2 mRNA Covid-19 vaccine through 6 months. N Engl J Med. 2021 Nov 4;385(19):1761–73. 10.1056/NEJMoa2110345 34525277 PMC8461570

[22] Al Kaabi N, Zhang Y, Xia S, Yang Y, Al Qahtani MM, Abdulrazzaq N, et al. Effect of 2 inactivated SARS-CoV-2 vaccines on symptomatic COVID-19 infection in adults: a randomized clinical trial. JAMA. 2021 Jul 6;326(1):35–45. 10.1001/jama.2021.8565 34037666 PMC8156175

[23] Coppeta L, Balbi O, Grattagliano Z, Mina GG, Pietroiusti A, Magrini A, et al. First dose of the BNT162b2 mRNA COVID-19 vaccine reduces symptom duration and viral clearance in healthcare workers. Vaccines (Basel). 2021 06 17;9(6):659. 10.3390/vaccines9060659 34204252 PMC8234325

[24] Antonelli M, Penfold RS, Merino J, Sudre CH, Molteni E, Berry S, et al. Risk factors and disease profile of post-vaccination SARS-CoV-2 infection in UK users of the COVID Symptom Study app: a prospective, community-based, nested, case-control study. Lancet Infect Dis. 2022 Jan;22(1):43–55. 10.1016/S1473-3099(21)00460-6 34480857 PMC8409907

[25] Pritchard E, Matthews PC, Stoesser N, Eyre DW, Gethings O, Vihta K-D, et al. Impact of vaccination on new SARS-CoV-2 infections in the United Kingdom. Nat Med. 2021 Aug;27(8):1370–8. 10.1038/s41591-021-01410-w 34108716 PMC8363500

[26] Leulseged TW, Hassen IS, Edo MG, Abebe DS, Maru EH, Zewde WC, et al. Duration of supplemental oxygen requirement and predictors in severe COVID-19 patients in Ethiopia: a survival analysis. Ethiop J Health Sci. 2021 Jul;31(4):699–708. 10.4314/ejhs.v31i4.3 34703168 PMC8512943

[27] Daher A, Balfanz P, Aetou M, Hartmann B, Müller-Wieland D, Müller T, et al. Clinical course of COVID-19 patients needing supplemental oxygen outside the intensive care unit. Sci Rep. 2021 01 26;11(1):2256. 10.1038/s41598-021-81444-9 33500431 PMC7838409

[28] Brouqui P, Amrane S, Million M, Cortaredona S, Parola P, Lagier JC, et al. Asymptomatic hypoxia in COVID-19 is associated with poor outcome. Int J Infect Dis. 2021 Jan;102:233–8. 10.1016/j.ijid.2020.10.067 33130200 PMC7604151

[29] Couzin-Frankel J. The mystery of the pandemic’s ‘happy hypoxia’. Science. 2020 May 1;368(6490):455–6. 10.1126/science.368.6490.455 32355007

